# Supplementary material for: Species-specific effects of passive warming in an Antarctic moss system
Source: R Soc Open Sci. 2019 Nov 13;6(11):190744. doi: 10.1098/rsos.190744 (PMC6894601; doi:10.1098/rsos.190744)
Supplement: OTC warming experiment on Juan Carlos Point, King George Island, South Shetland Islands, Antarctica [file rsos190744supp1.docx]

**OTC warming experiment on Juan Carlos Point, King George Island, South Shetland Islands, Antarctica**

**Supplementary Figure 1 A)** OTC warming experiment on Juan Carlos Point, King George Island, Antarctica. **B)** Dissected archegonia of female *Polytrichastrum alpinum* from control plot. **C)** The dominant terrestrial plant cover (including *Polytrichastrum alpinum* and *Sanionia georgicouncinata*), on King George Island.
